# A gonococcal homologue of meningococcal γ-glutamyl transpeptidase gene is a new type of bacterial pseudogene that is transcriptionally active but phenotypically silent

**DOI:** 10.1186/1471-2180-5-56

**Published:** 2005-10-04

**Authors:** Hideyuki Takahashi, Haruo Watanabe

**Affiliations:** 1 Department of Bacteriology, National Institute of Infectious Diseases, Tokyo, Japan

## Abstract

**Background:**

It has been speculated that the γ-glutamyl transpeptidase (*ggt*) gene is present only in *Neisseria meningitidis *and not among related species such as *Neisseria gonorrhoeae *and *Neisseria lactamica*, because *N. meningitidis *is the only bacterium with GGT activity. However, nucleotide sequences highly homologous to the meningococcal *ggt *gene were found in the genomes of *N. gonorrhoeae *isolates.

**Results:**

The gonococcal homologue (*ggt *gonococcal homologue; *ggh*) was analyzed. The nucleotide sequence of the *ggh *gene was approximately 95 % identical to that of the meningococcal *ggt *gene. An open reading frame in the *ggh *gene was disrupted by an ochre mutation and frameshift mutations induced by a 7-base deletion, but the amino acid sequences deduced from the artificially corrected *ggh *nucleotide sequences were approximately 97 % identical to that of the meningococcal *ggt *gene. The analyses of the sequences flanking the *ggt *and *ggh *genes revealed that both genes were localized in a common DNA region containing the *fbp-ggt *(or *ggh)-glyA-opcA-dedA-abcZ *gene cluster. The expression of the *ggh *RNA could be detected by dot blot, RT-PCR and primer extension analyses. Moreover, the truncated form of *ggh-*translational product was also found in some of the gonococcal isolates.

**Conclusion:**

This study has shown that the gonococcal *ggh *gene is a pseudogene of the meningococcal *ggt *gene, which can also be designated as Ψ*ggt*. The gonococcal *ggh *(Ψ*ggt*) gene is the first identified bacterial pseudogene that is transcriptionally active but phenotypically silent.

## Background

Two members of the gram-negative diplococci, *Neisseria meningitidis *and *Neisseria gonorrhoeae*, are particularly associated with pathological infections. *N. meningitidis *is specialized for the mucosa of the nasopharynx and causes meningitis and septicemia. *N. gonorrhoeae *is adapted for the mucosa of the urogenital tract and causes gonorrhoea and pelvic inflammatory diseases. Both species colonize only humans and share a great deal of relatedness at the nucleotide level [1]. This high degree of relatedness is reflected in the many common genetic, biochemical and antigenic features of the two bacteria.

γ-Glutamyl transpeptidase (also called γ-glutamyl aminopeptidase) (EC2.3.2.2; GGT) catalyzes the hydrolysis of γ-glutamyl compounds, and is found in a variety of bacteria such as *Escherichia coli *[2] and *Helicobacter pylori *[3,4]. To distinguish *N. meningitidis *from *N. gonorrhoeae*, GGT activity is used as one of the identification markers for *N. meningitidis *because *N. meningitidis *is positive for this activity but *N. gonorrhoeae *and related species, e.g., *Neisseria lactamica*, are not [5]. In fact, the detection of GGT activity is applied for the identification of *N. meningitidis *in the Gonochek II enzymatic identification system (E-Y Laboratories Inc., U.S.A.) [6-9]. From these empirical facts, it was believed that the gene encoding for GGT should exist only in *N. meningitidis*, but this has not been proven yet [3].

Recent remarkable progress in the sequencing of various genomes has led to the detection of nucleotide sequences that appear to be phenotypically silent, termed pseudogenes. The pseudogenes are defined as DNA sequences of formerly functional genes rendered nonfunctional by mutations and usually identified by their disrupted open reading frames (ORFs). Pseudogenes have been identified in a variety of eukaryotes, including insects [10], plants [11], and particularly vertebrates [10,12], but are relatively few in the bacterial genomes. Notable exceptions are intracellular bacterial parasites such as *Rickettsia prowazekii *and *Mycobacterium leprae *[13], which seem to have lost many genes due to obtaining nutritional supplies from the host cells. Cryptic genes such as the *cel *operon in *E. coli *[14-16] and the flagellar master operon in the genus *Shigella *[17-19] seem to be a kind of pseudogenes, but are different from pseudogenes because the cryptic genes completely retain intact ORFs, which can be occasionally activated by rare genetic events such as mutation, recombination, insertion of elements. As a whole, compared to the pseudogenes in eukaryotes, relatively few pseudogenes have been reported in bacterial genomes [20].

In this study, a gonococcal *ggh *gene, which is highly homologous to the meningococcal *ggt *gene, was found to be pseudogene. Sequence analyses of the flanking regions of both the *ggt *and *ggh *genes suggest that both genes were derived from a gene in a common ancestor, and subsequently diversified.

## Results

### The gonococcal *ggh *gene was highly homologous to the meningococcal *ggt *gene

**Figure 1:**
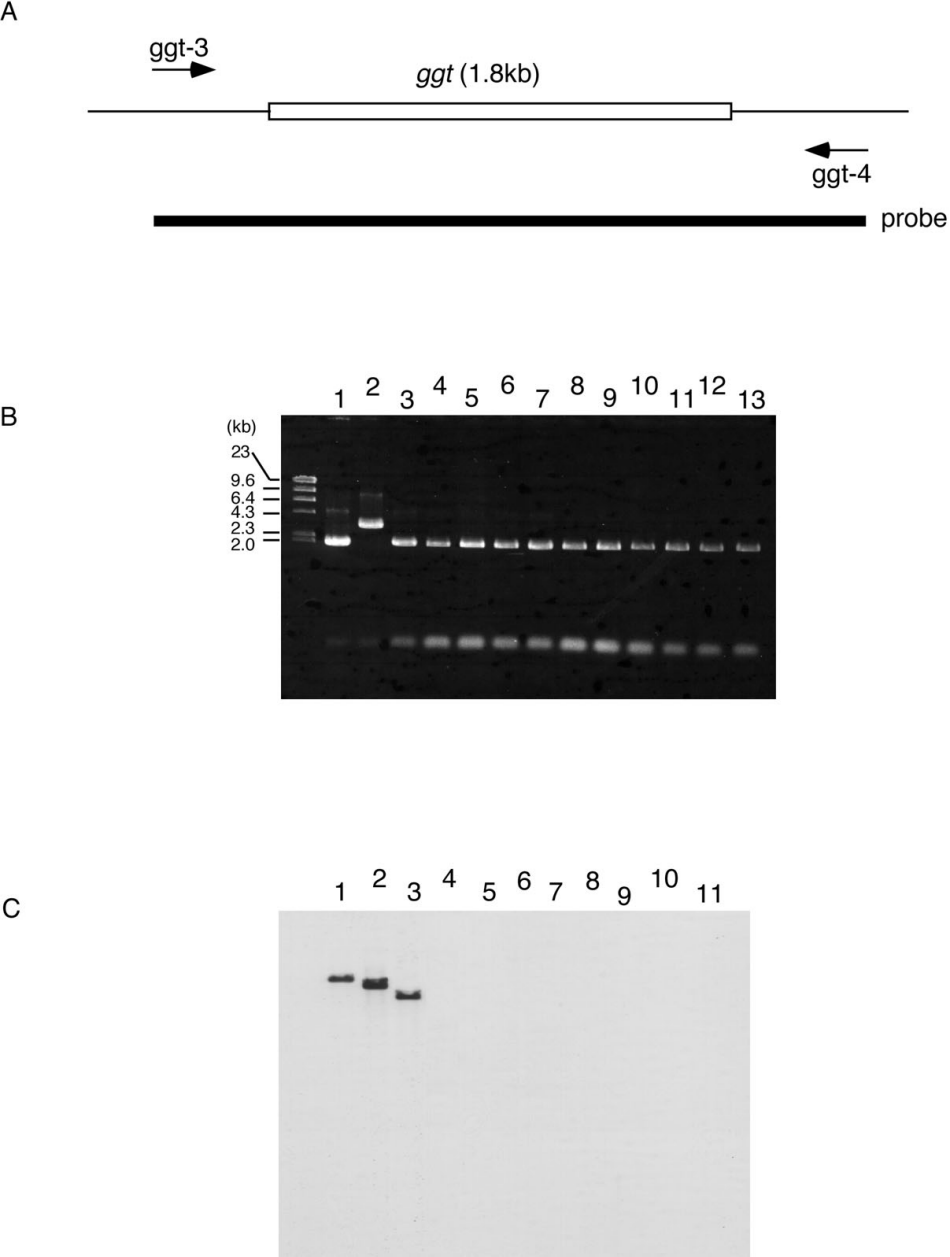
**The presence of a meningococcal *ggt *gene homologue in *N. gonorrhoeae*. **A. A schematic diagram showing the position of the set of primers used for the detection of *ggt *gene homologues. The black bar shows the region of the DNA probe used for Southern blotting in panel C. B. Amplification of gonococcal *ggh *gene by PCR. The genomic DNAs of neisserial species used for PCR were as follows: lane 1, H44/76 (*N. meningitidis ggt*^+^); lane 2, NIID113 (*N. meningitidis ggt::*IS) [49]; lane 3–13, ATCC49226, NIID54, NIID102, NIID103, NIID104, NIID105, NIID106, NIID107, NIID108, NIID109, NIID111 (*N. gonorrhoeae*). C. Southern blotting using the meningococcal *ggt *gene as a probe. Two micrograms of purified chromosomal DNA digested with *Cla*I were subjected to this analysis. Lane 1, H44/76 (*N. meningitidis ggt*^+^); lane 2, NIID113 (*N. meningitidis ggt::*IS) [49]; lane 3, NIID54 (*N. gonorrhoeae*); lane 4, ATCC23970 (*N. lactamica*); lane 5, ATCC13120 (*N. flavescens*); lane 6, ATCC14686 (*N. denitrificans*); lane 7, ATCC25295 (*N. elongata*); lane 8, ATCC14687 (*N. canis*); lane 9, ATCC14685 (*N. cinerea*); lane 10, NIID16 (*N. mucosa*); lane 11, NIID17 (*N. sicca*).

Since GGT activity was detected only in *N. meningitidis *among the related species, it was speculated that the corresponding gene also existed only in *N. meningitidis*. However, by BLAST search, the nucleotide sequences highly homologous to the meningococcal *ggt *gene were found in the genome of *N. gonorrhoeae *FA1090 [GenBank:NC_002946]. The overall nucleotide sequence of the meningococcal *ggt *homologue was approximately 95 % identical to that of the meningococcal *ggt *gene (data not shown and additional file). Eleven *N. gonorrhoeae *clinical strains were analyzed by PCR, and the corresponding DNA fragments were amplified in all of these strains (Figure 1B), indicating that this gene was generally present in *N. gonorrhoeae*. To analyze whether *ggt *homologues existed in the genomes of the other neisserial strains, Southern blotting was performed (Figure 1C). DNA fragments that hybridized with the meningococcal *ggt *gene were found in the meningococcal and gonococcal genomes (Figure 1C lanes 1–3) but not in the other neisserial genomes (Figure 1C lanes 4–11). These results suggested that the meningococcal *ggt *homologue was present only in *N. gonorrhoeae *among the neisserial species examined. The putative gene in *N. gonorrhoeae *was named *ggh *(*ggt *gonococcal homologue).

### Variations of the nucleotide sequences of *ggh *genes among clinical isolates

**Figure 2:**
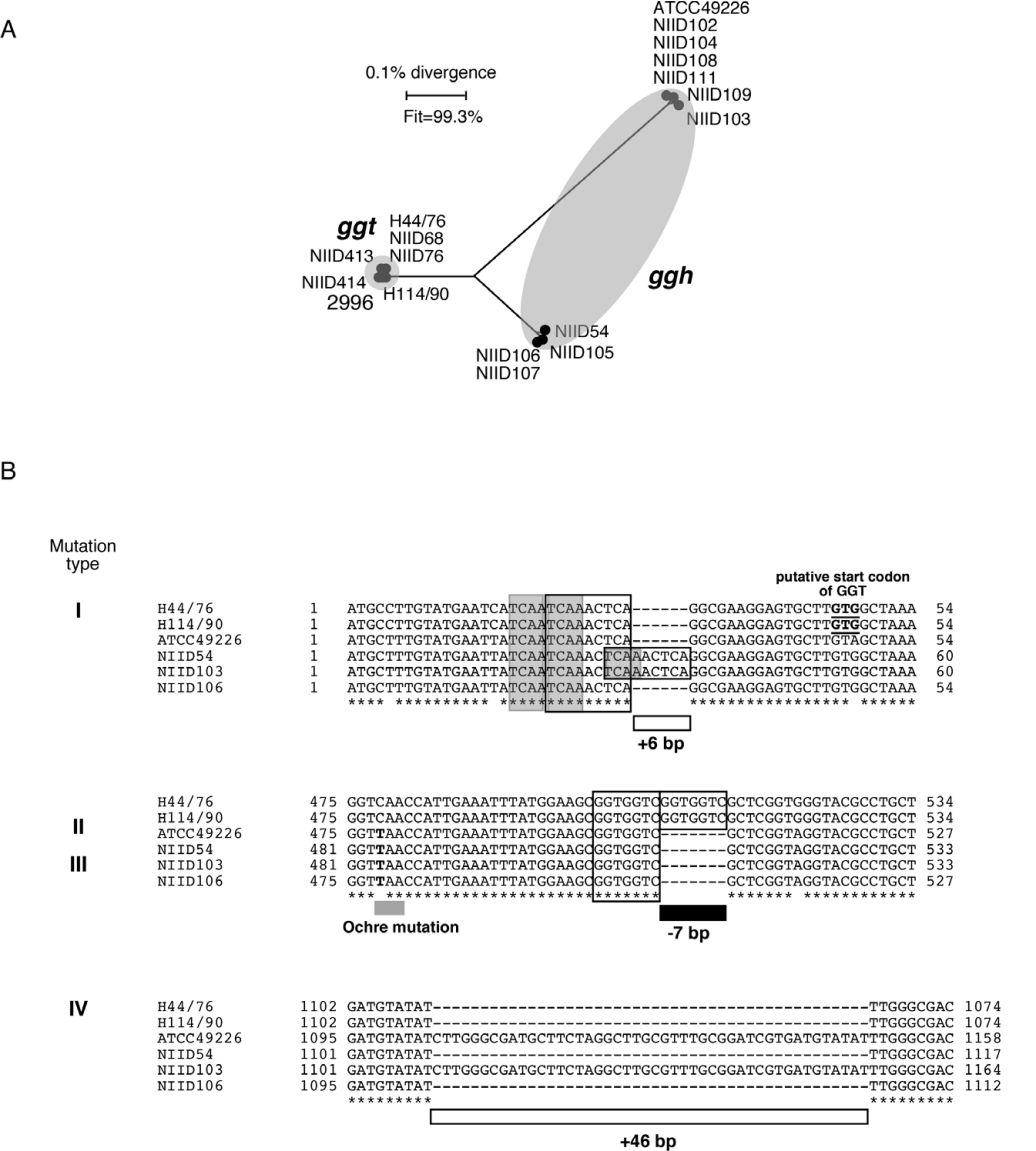
A. Split graph showing the relationships among *ggt *genes in 7 meningococcal strains and *ggh *genes in 11 gonococcal strains. The sequence data have been submitted to the DDBJ/EMBL/GenBank Databases under the following Accession Numbers:*N. meningitidis *strains H44/76 [DDBJ:AB089320], H114/90 [DDBJ:AB177989], 2996 [DDBJ:AB177990], NIID68 [DDBJ:AB177991], NIID76 [DDBJ:AB177992], NIID413 [DDBJ:AB177993] and NIID414 [DDBJ:AB177994]; *N. gonorrhoeae *strains ATCC49226 [DDBJ:AB175023], NIID54 [DDBJ:AB175024], NIID102 [DDBJ:AB193248], NIID103 [DDBJ:AB175025], NIID104 [DDBJ:AB175026], NIID105 [DDBJ:AB193249], NIID106 [DDBJ:AB175027], NIID107 [DDBJ:AB175028], NIID108 [DDBJ:AB193250], NIID109 [DDBJ:AB194328], NIID111 [DDBJ:AB193251]. The scale bar represents uncorrected distances, and a fit parameter is also shown. B. Alignment of the nucleotide sequences containing 4 kinds of differences in the *ggt *and *ggh *genes of *N. meningitidis *strains H44/76 and H114/90; *N. gonorrhoeae *strains ATCC49226, NIID54, NIID103 and NIID106, respectively. Sequence identity is represented as *, polymorphism among the sequences of the 6 strains is indicated by the appropriate letter, and the absence of a base is shown by a hyphen (-). The nucleotide substitution for an ochre (Type II) mutation is shown in bold. Boxes at the Type I and Type III mutations indicate the tandem repeat. The tetranucleotide repeat in type I is also shown as a gray box. The newly deduced start codon of the meningococcal GGT is shown in underlined bold.

To characterize the gonococcal *ggh *gene, the *ggh *genes were amplified from the chromosomal DNA of 11 *N. gonorrhoeae *strains and sequenced. The nucleotide sequences of the *ggt *genes from 7 *N. meningitidis *strains and of the *ggh *genes from the 11 *N. gonorrhoeae *strains were aligned, and the distance matrix calculated from these data was displayed as a phylogenetic tree (Figure 2A). The results revealed that the nucleotide sequences of the gonococcal *ggh *genes were more divergent than those of the meningococcal *ggt *genes (Figure 2A). Alignment of the nucleotide sequences of the 11 gonococcal *ggh *genes also showed that the mutations in the *ggh *gene consisted of the following four polymorphisms: 1) a 6-base insertion (named Type I), 2) an ochre mutation (Type II), 3) a 7-base deletion (Type III), 4) a 46-base insertion (Type IV) (Figure 2B and Table 2). In addition, all of the 11 *ggh *genes had one-nucleotide substitutions compared to the *ggt *genes in the same 25 sites (Figure 2B and additional file), with only 2 exceptions: a one-nucleotide variation in NIID109 (48^th ^base A to G) and another in NIID105 (213^th ^base G to A) (Table 2). The one-base substitutions in the common sites of the *ggh *genes strongly suggested that reconstruction of the *ggh *gene would have occurred at an early stage after speciation (See Discussion).

### Putative amino acid sequences in hypothetical coding region of the *ggh *genes

**Figure 3:**
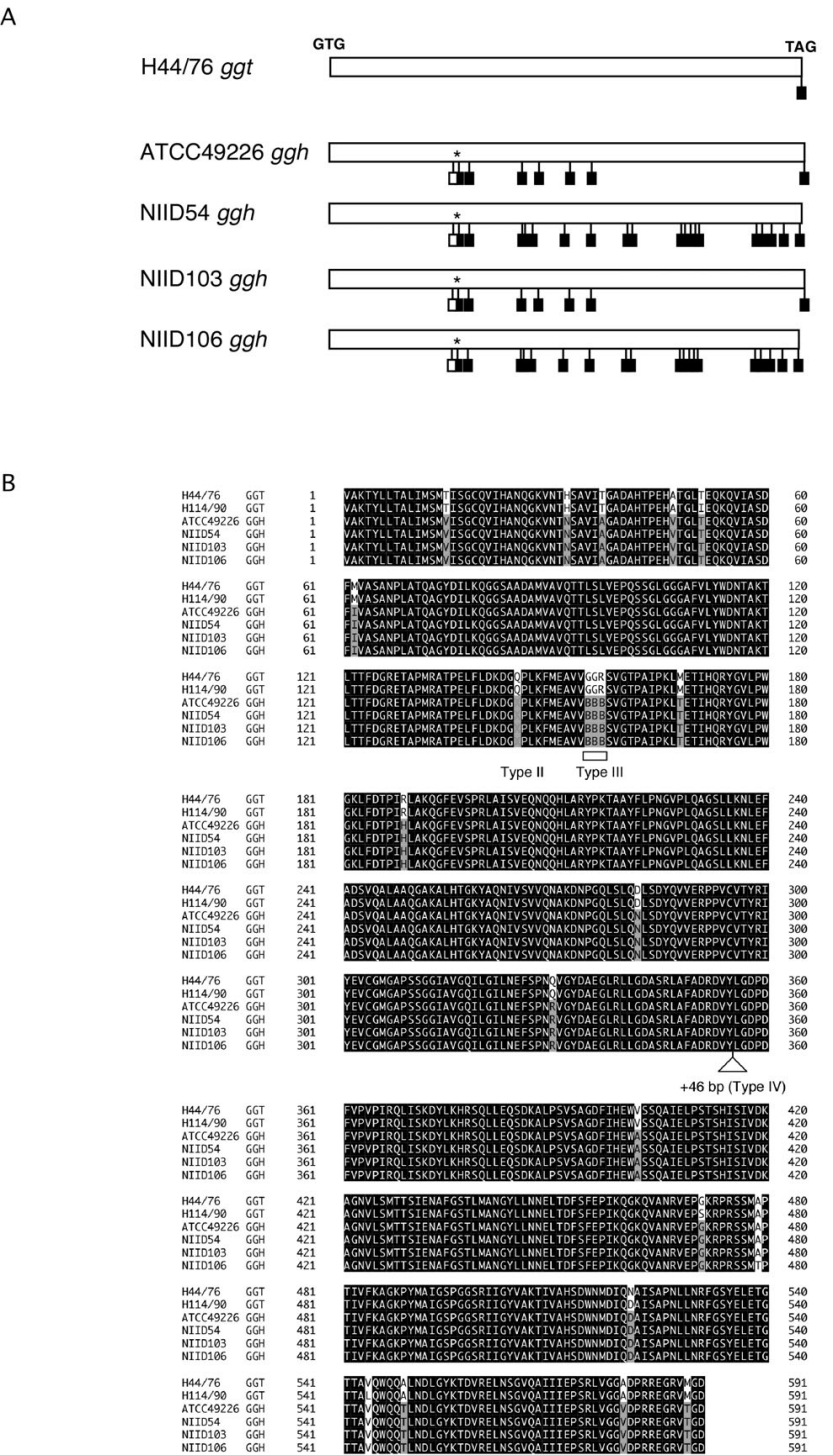
A. Positions of stop codons in the hypothetical ORF in the *ggh *gene. Black squares indicate the positions of stop codons and white squares indicate the ochre (Type II) mutation shown in Figure 2B. * indicates the position of the 7-bp deletion (Type III) mutation in the *ggh *gene. B. Putative translated products from the corrected nucleotide sequences of the *ggh *genes. The Type IV insertional mutation shown in Figure 2B is removed and the site of Type III deletion is replaced by the letter B. The site of the ochre (Type II) mutation is shown as X. The identical amino acid sequences between *N. meningitidis *and *N. gonorrhoeae *are shown in a black box and amino acid sequences common to only *N. gonorrhoeae *are shown in a gray box.

Due to the ochre (Type II) and 7-base deletion (Type III) mutations, the ORF in each *ggh *gene was completely disrupted by the formation of 8 or 20 stop codons (Figure 3A). In fact, none of the gonococcal isolates showed any GGT activity (data not shown and [5,9]), indicating that there was no expression of functional GGT-like protein in *N. gonorrhoeae*. All of these results showed that the gonococcal *ggh *gene was a pseudogene of the functional *ggt *gene in *N. meningitidis*. On the other hand, if the two types of mutations (Types II and III) in the *ggh *genes were artificially corrected, the hypothetical amino acid sequences were approximately 97 % identical to those of the meningococcal *ggt *genes and were highly conserved among the gonococcal *ggh *genes (Figure 3B). This result also supported the idea that the *ggh *and *ggt *genes were derived from a common ancestral gene and that the translational inactivation of the gonococcal *ggh *gene was solely due to the ochre (Type II) and the frame-shift mutations caused by the 7-base deletion (Type III).

### The genetic organization of the *ggt*- and *ggh*-flanking regions in the genomes of *N. meningitidis *and *N. gonorrhoeae*

**Figure 4:**
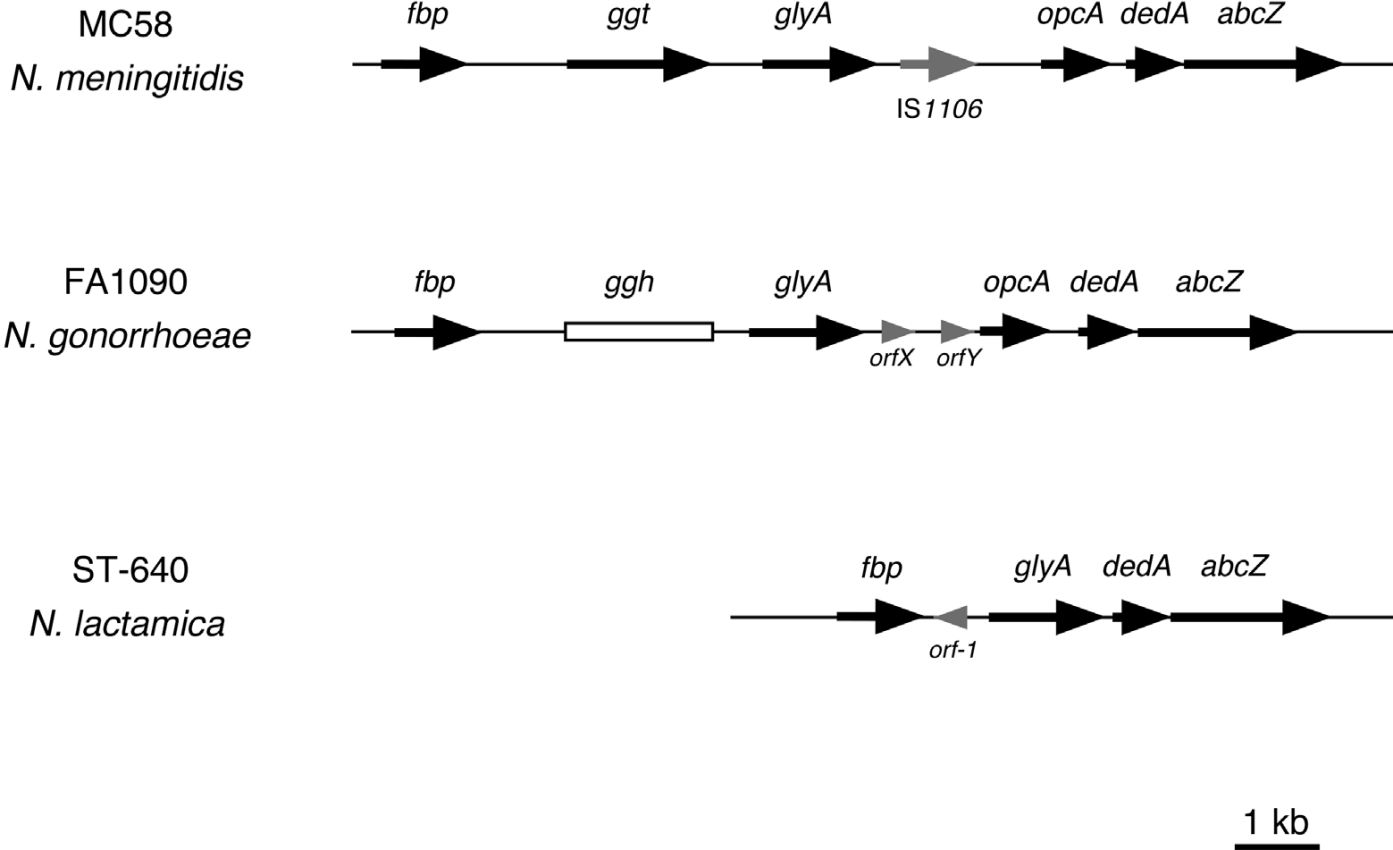
Genetic organization of the genes around the *ggt *and *ggh *genes in *N. meningitidis *strain MC58 [21], *N. gonorrhoeae *strain FA1090 [47] and *N. lactamica *strain ST-640 [48]. Black arrows and the open bar indicate coding and non-coding genes, respectively.

By using the information in the database for *N. meningitidis *strain MC58 [21], *N. gonorrhoeae *strain FA1090 and *N. lactamica *(the neisserial species most closely related to the above two species) ST-640 strain, the flanking regions of the meningococcal *ggt *and the gonococcal *ggh *genes were further analyzed. The *ggt *and *ggh *genes were both localized in the identical gene cluster of *fbp-ggt *(or *ggh)-glyA-opcA-dedA-abcZ *in the genomes of *N. meningitidis *and *N. gonorrhoeae*, respectively (Figure 4). The *fbp-glyA-dedA-abcZ *locus was also found in the genome of *N. lactamica *but lacked the *ggt *and *opcA *homologues (Figure 4). This highly conserved genetic organization implied that a DNA island containing an original *ggt *gene was first incorporated into an ancestor's genome of the above three species and subsequently diversified after the speciation (see Discussion).

### The expression of the *ggh *gene in *N. gonorrhoeae*

**Figure 5:**
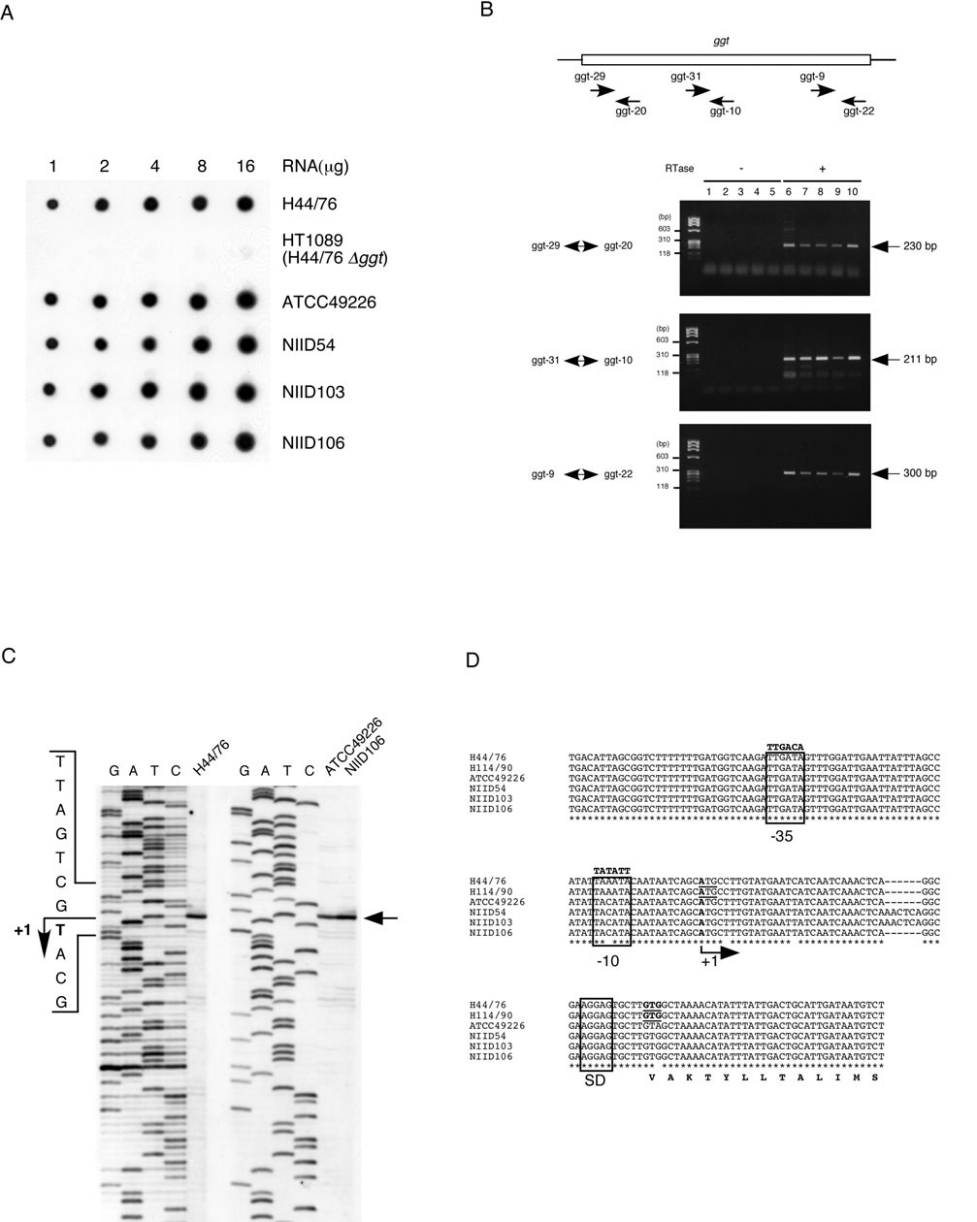
**Transcriptional expression of the *ggh *genes. **A. Dot blot analysis using the meningococcal *ggt *gene as a probe. One to 16 micrograms of RNAs isolated from H44/76, HT1089 (H44/76 *Δggt::spc*) [39], ATCC49226, NIID54, NIID103 and NIID106 were subjected to this analysis. B. RT-PCR to detect the transcripts of the gonococcal *ggh *genes. The schematic figure in the box depicts the position of the primers used in this experiment (see Table 1). RT-PCR was performed without reverse transcriptase (RTase) (lanes 1 to 5) or with RTase (lanes 6 to 10). Lanes 1 and 6, H44/76 (*N. meningitidis*); lanes 2 and 7, ATCC49226 (*N. gonorrhoeae*); lanes 3 and 8, NIID54 (*N. gonorrhoeae*); lanes 4 and 9, NIID103 (*N. gonorrhoeae*); lanes 5 and 10, NIID106 (*N. gonorrhoeae*). The marker in the left-most lane is *φ *X174 DNA digested with *Hae*III. Primer sets used for RT-PCR are shown on the left side, and the corresponding PCR products are indicated by arrows on the right side. C. Primer extension analysis to detect the transcriptional start point of the *ggt *and *ggh *genes. Total RNA extracted from H44/76 (*N. meningitidis*), ATCC49226 and NIID106 (*N. gonorrhoeae*) was used for the primer extension with AMV reverse transcriptase XL and biotin-labeled oligonucleotide ggt-ext-2. The arrow on the right side indicates the transcriptional start site. D. Alignment of the nucleotide sequences of the upstream regions of the *ggt *and *ggh *genes. The sequence data have been deposited in the DDBJ/EMBL/GenBank Databases under the following Accession Numbers:*N. meningitidis *strains H44/76 [DDBJ:AB193252], H114/90 [DDBJ:AB193253], *N. gonorrhoeae *strains ATCC49226 [DDBJ:AB193254], NIID54 [DDBJ:AB193255], NIID103 [DDBJ:AB193296], NIID106 [DDBJ:AB193256]. An identical nucleotide is represented as *. The transcriptional start site is shown in bold as +1. The putative -35, -10 elements and Shine-Dalgarno sequence (SD) are depicted in the box, and the ideal -35 and -10 nucleotide sequences are shown above the boxes. The previously predicted start codon (ATG) [25] and newly predicted start codon (GTG) of the meningococcal {\it ggt} gene are underlined. The amino acid sequence deduced from the putative start codon GTG (shown in bold) in the meningococcal {\it ggt} gene is also shown under the corresponding nucleotide sequences.

The hitherto identified bacterial pseudogenes are not expressed transcriptionally or translationally [20,22-24]. To examine the *ggh *transcriptional expression, dot blot analysis was first performed and RNA that hybridized with the *ggt *probe was detected in the total RNAs of 4 *N. gonorrhoeae *strains (Figure 5A). The transcriptional expression was also confirmed by RT-PCR, and the products were amplified with all 3 sets of primers from total RNA of all 4 *N. gonorrhoeae *strains tested (Figure 5B). These results strongly suggested that the full-length *ggh *gene transcript was expressed in *N. gonorrhoeae*. Primer extension analysis further revealed that the gonococcal *ggh *RNA was transcribed from the same starting point as the meningococcal *ggt *mRNA (Figure 5C and 5D). All of these results indicated that the gonococcal *ggh *gene was transcriptionally active though it was a pseudogene.

**Figure 6:**
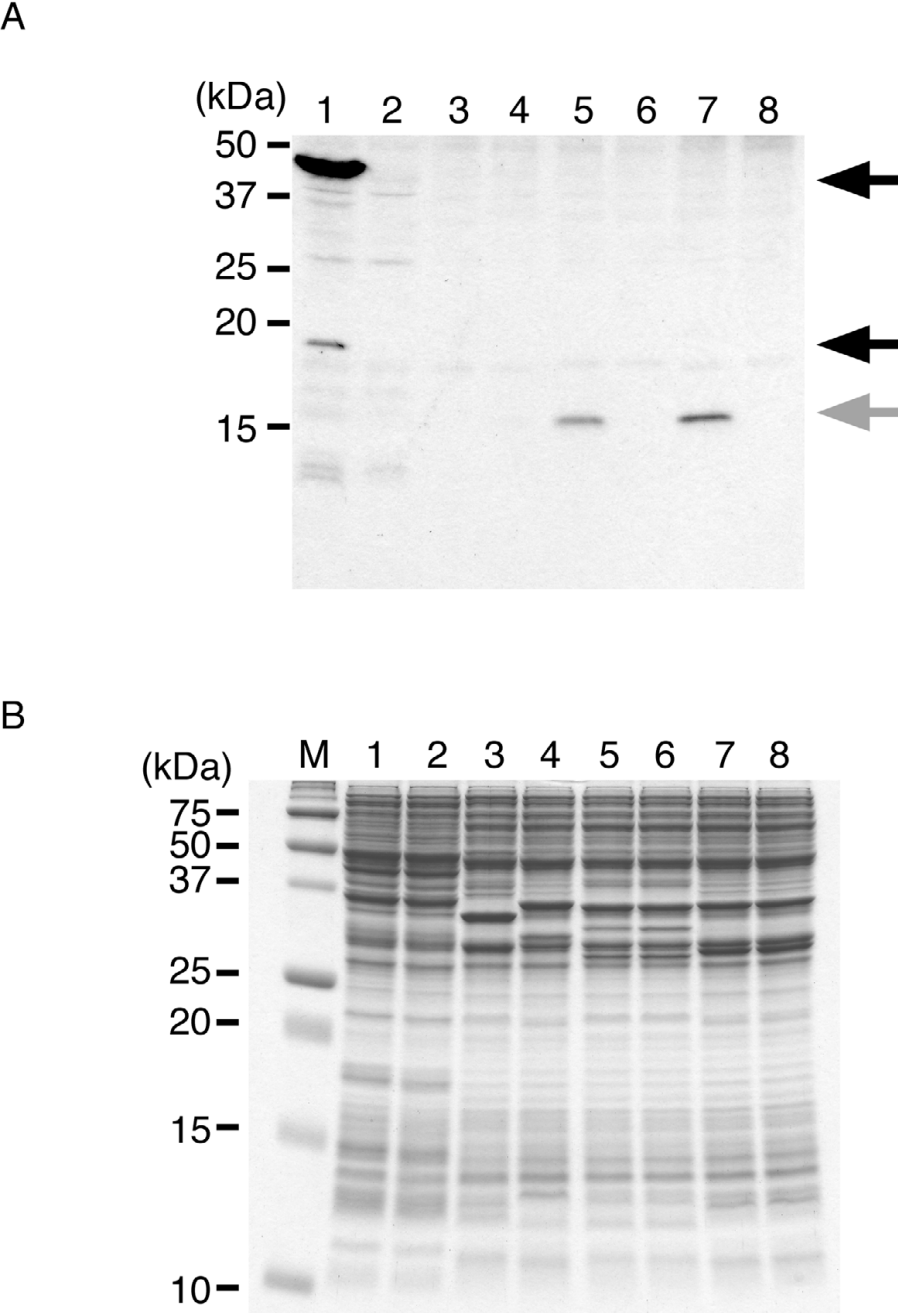
**Western blotting with anti-meningococcal GGT rabbit antiserum [25] (A) and Coomassie Brilliant Blue staining (B) of the whole cell extracts after SDS-PAGE. **Bacterial whole cell extracts equivalent to 0.025 OD_600 _were analyzed. Lane 1, *N. meningitidis *strain H44/76; lane 2, HT1089 (H44/76 *Δggt::spc*); lane 3, ATCC49226 (*N. gonorrhoeae*); lane 4, NIID54 (*N. gonorrhoeae*); lane 5, NIID103 (*N. gonorrhoeae*); lane 6, HT1195 (NIID103 *Δggh::spc*); lane 7, NIID106 (*N. gonorrhoeae*); lane 8, HT1196 (NIID106 *Δggh::spc*). Black arrows show the bands corresponding to the processed small and large subunits of meningococcal GGT and the gray arrow indicates the 15-kDa band corresponding to the truncated protein product of the gonococcal *ggh *gene. M stands for molecular weight marker.

To further study the translational expression of the truncated GGT-like protein in *N. gonorrhoeae*, Western blotting was performed with anti-meningococcal GGT rabbit antiserum [25]. When the same amounts of the whole cell extracts were analyzed (Figure 6B), approximately 15-kDa bands were detected in the extracts of NIID103 and NIID106 *N. gonorrhoeae *strains (Figure 6A lanes 5 and 7). Because the 15-kDa protein was not observed in the *Δggh *background of NIID103 and NIID106 (Figure 6A lanes 6 and 8), the 15-kDa protein was thought to be the *ggh *gene product whose translation was terminated at the 145th codon (Figure 3B). However, the 15-kDa protein was not found in the extracts of the ATCC49226 and NIID54 *N. gonorrhoeae *strains (Figure 6A, lanes 3 and 4) and seemed not to be expressed in any of the other gonococcal strains (see Discussion).

## Discussion

In this study, it was shown that the gonococcal *ggh *gene is a member of bacterial pseudogenes, and is transcribed but not properly translated so that active *ggh *protein product is not produced. *11Fh-mtTFA *[26], *OsMu4-2 *[27], *NA88-A *[28], *Makorin1-p1 *[29], *Dnm3a2 *[30] and *pseudoNOS *[31] genes are known to be transcriptionally active eukaryotic pseudogenes. In neisseriae, the gonococcal *porA *and Ψ*opcB *genes have been reported as neisserial pseudogenes [1,32,33]. The *porA *pseudogene contains mutations in the promoter and the coding regions, and is not translated [24]. While some hypothetical bacterial pseudogenes with repetitive runs of A and T are speculated to be potentially expressed by transcriptional slippage [34], the expression, including the transcription, has not been proven yet. To our knowledge, the gonococcal *ggh *gene is the first identified bacterial pseudogene that is transcriptionally active.

The 15-kDa derivative of the putative *ggh *protein product is detected in the NIID103 and NIID106 *N. gonorrhoeae *strains but not in the ATCC49226 and NIID54 *N. gonorrhoeae *strains (Figure 6A). Since the predicted amino acid sequences of the putative 15-kDa proteins seem to be similar among the 4 gonococcal strains, the reason why the 15-kDa protein was not detected in ATCC49226 and NIID54 is not clear. The 15-kDa protein might be degraded in ATCC49226 and NIID54 backgrounds but not in NIID103 and NIID106 backgrounds. It seems unlikely that the 15-kDa protein encoded by the *ggh *gene has an essential function for *N. gonorrhoeae *because the 15-kDa protein is not always detected in any of gonococcal strains (Figure 6A).

Why does the gonococcal *ggh *gene still retain the transcriptional activity? There are some examples in which RNAs transcribed from pseudogenes have some biological functions: antisense RNA expressed from the *pseudoNOS *gene hybridizes with *nNOS *(nitric oxide synthase) mRNA, resulting in the suppression of the nNOS gene expression in the neurons of the snail *Lymnaea stagnalis *[31]. RNA of *Makorin1-1p*, a pseudogene of *Makorin1*, regulates the *Makorin1 *mRNA stability, which is important for the correct formation of the kidneys and bone in mice [29]. Some eukaryotic genes may be duplicated and one of the plural genes may be subsequently reconstructed due to its redundancy, resulting in a pseudogene. However, since a bacterial pseudogene generally does not have a functional counterpart (wild-type gene) in a single organism, the *ggh *RNA does not seem to have the same kind of biological function as the *pseudoNOS *and *Makorin1-1p *genes. In fact, we could not find any prominent phenotype for a *Δggh *gonococcal mutant (unpublished data). However, we cannot exclude the possibility that the *ggh *RNA has some biological function(s) in other milieus such as the urogenital tract and further analyses will be required to address this possibility.

The *ggt *and *ggh *genes are located in the *fbp-ggt (ggh)-glyA-opcA-dedA-abcZ *common gene cluster in the genomes of both *N. meningitidis *and *N. gonorrhoeae *(Figure 4) [23]. The genome of *N. lactamica *lacks *ggt *and *opcA *homologues in the *fbp-glyA-dedA-abcZ *locus (Figure 4 and [23]). It would not be the result of chance that the two *ggt *(or *ggh*) and *opcA *genes are located in the same respective sites of the *fbp-glyA-dedA-abcZ *gene locus of both *N. meningitidis *and *N. gonorrhoeae *but not in that of *N. lactamica*. Moreover, it is also unlikely that a nonfunctional *ggh *gene was horizontally transferred into the gonococcal *fbp-glyA-opcA-dedA-abcZ *gene cluster since such a pseudogene could not have been sustained due to the lack of selection [23]. Therefore, it seems more probable that the *fbp-ggt-glyA-opcA-dedA-abcZ *gene cluster was present in an ancestor of the three neisserial species, and has been subsequently diversified independently among the three species, as shown for the *opcA *gene [23]. During the diversification, the meningococcal *ggt *gene has been maintained in an active state while the gonococcal *ggh *gene has been reconstructed by insertion, deletion and substitutions, resulting in the translational inactivation. In *N. lactamica*, the *ggt *and *opcA *homologues might have been lost because of their dispensability for *N. lactamica *(see below).

It is also interesting that the *ggh *gene has not been fully deleted from the gonococcal genome. The kinds and sites of mutations in the *ggh *genes are relatively few and highly conserved, respectively, among the gonococcal isolates (Figure 2B, Table 2 and additional file). It is also noted that, while in general the RNA polymerase-binding sites and SD regions of pseudogenes are highly degraded, there are also a few exceptions in species such as *Y. pestis *that could have emerged in recent evolutionary times [35]. Since the ribosome-binding sites (Shine-Dalgarno regions) of the gonococcal *ggh *gene are identical to those of the meningococcal *ggt *gene and the RNA polymerase-binding sites are almost completely conserved (with a one-nucleotide difference) (Figure 5D), it is speculated that the reconstruction of the *ggh *gene may have occurred in relatively recent evolutionary times. From the evolutional viewpoint, the drastic deletion of the approximately 2-kb DNA region containing the *ggh *gene in *N. gonorrhoeae *may not have been likely to occur in such a short period. Alternatively, deletion of the 2-kb DNA region may not be more advantageous for gonococcal evolution than reconstruction involving short deletions, insertions and substitutions.

The maintenance of a functional *ggt *gene in *N. meningitidis *would have some advantages for its survival. *N. meningitidis *causes meningitis, which is due to the meningococcal invasion into the human central nervous system, including cerebrospinal fluid (CSF) [36-38]. It has been shown that meningococcal GGT has a physiological function of acquiring cysteine from environmental γ-glutamyl-cysteinyl peptides under cysteine-limited environments such as the CSF [39]. Almost all (98.8 %) meningococcal isolates from humans are positive for GGT activity [40]. All of these results suggest that the GGT activity is important for *N. meningitidis *but not for *N. gonorrhoeae*. The dispensability of GGT activity for *N. gonorrhoeae *seems to be consistent with the fact that a cysteine-limited milieu such as the CSF in humans is not a natural gonococcal habitat. However, it is not very likely that the milieu of CSF exerts selective pressure for an active *ggt *gene because human CSF is not a relevant milieu for human-to-human spread of meningococcus [41]. We believe that the meningococcal GGT must have some unknown essential function(s) for *N. meningitidis *and further studies will elucidate the function(s).

## Conclusion

Our data on the *ggh *gene indicate that the *ggh *gene in *N. gonorrhoeae *is a pseudogene of the functional *ggt *gene in *N. meningitidis*. To our knowledge, the *ggh *gene is the first reported bacterial pseudogene that is transcriptionally active but phenotypically silent. Our findings may also contribute to understanding the speciation of *N. meningitidis *and *N. gonorrhoeae*.

## Methods

### Bacterial strains and growth conditions

Seven *N. meningitidis *strains (H44/76, H114/90, 2996, NIID68, NIID76, NIID413 and NIID414) were described in our previous reports [42,43]. Ten *N. gonorrhoeae *strains (NIID54, NIID102, NIID103, NIID104, NIID105, NIID106, NIID107, NIID108, NIID109 and NIID111), one *N. mucosa *strain (NIID16) and one *N. sicca *strain (NIID17) were clinical isolates donated by T. Kuroki and Y. Watanabe. All of the clinical strains were mutually independent: they were isolated in different periods and from different persons who lived in different areas of Japan. The following 7 neisserial strains were obtained from ATCC (species / ATCC no.): *N. gonorrhoeae / *ATCC49226; *N. lactamica */ ATCC23970; *N. flavescence */ ATCC13120; *N. denitrificans */ ATCC14686; *N. elongata */ ATCC25295; *N. canis / *ATCC14687; *N. cinerea / *ATCC14685. All of the strains were stored by the gelatin disc method [42] and cultivated on GC agar (Becton-Dickinson) supplemented with 1 % IsovitaleX enrichment (Becton-Dickinson) at 37°C in 5 % CO_2_, or in GC broth (1.5 % proteose-peptone, 0.5 % NaCl, 0.05 % soluble starch, 0.1 % K_2_HPO_4_, 0.4 % KH_2_PO_4_, 1 % IsoVitaleX, 5 mM NaHCO_3 _10 mM MgCl_2_) at 37°C with shaking.

### Isolation of chromosomal DNA, PCR, Southern blotting and dot blotting

Isolation of chromosomal DNA, PCR, Southern blotting and dot blotting were performed as described in our previous report [43].

### Nucleotide sequence determination and analyses

The *ggt *and *ggh *genes were amplified with a set of primers (ggt-3 and ggt-4) by PCR, and the resulting products were purified using High Pure PCR Product Purification Kit (Roche) as templates for sequencing. The sequencing was performed as described previously [25]. Primers used for sequencing are listed in Table 1. Raw data from the ABI sequencer were assembled with the program DNASIS ver. 3.2 (HITACHI, Japan). The sequence alignment was performed with GENETYX-MAC ver.11 (GENETYX, Japan). Phylogenetic analyses were performed by constructing a distance matrix of nucleotide mismatches using the web site of the Belozersky Institute at Moscow State University [44] and visualized by Split decomposition analysis with the program SPLITSTREE, version 3.2 [45].

### Construction of *Δggh::spc N. gonorrhoeae *mutants

HT1195 (NIID103 *Δggh::spc*) and HT1196 (NIID106 *Δggh::spc*), in which a spectinomycin resistance gene (*spc*) was inserted into the *ggh *gene, were constructed as follows: A 2-kb fragment containing the *ggh *gene of NIID103 or NIID106 was amplified by PCR and cloned in the *Sma*I site of pUC18 (Takara Bio) to construct pHT412 or pHT413, respectively. A blunted 1-kb fragment containing the *spc *gene [25] was inserted into the *Eco*RV sites of pHT412 and pHT413, respectively. The *Eco*RV sites are located at 277 bp and 1642 bp (NIID103 [DDBJ:AB175025]) and at 271 bp and 1590 bp (NIID106 [DDBJ:AB175027]) downstream from the transcriptional start point of each *ggh *gene, respectively (see Figure 5D). The resulting plasmids were named pHT414 and pHT415, respectively. Five hundred nanograms of the plasmids linearized by digestion with *Eco*RI were transformed into NIID103 and NIID106, respectively, as described previously [43]. Spectinomycin-resistant clones were selected on GC agar plates containing 75 μg/ml spectinomycin. The resulting mutants were isolated as *Δggh::spc *NIID103 (HT1195), and *Δggh::spc *NIID106 (HT1196), respectively. The allelic exchange was confirmed by PCR and Southern blotting.

### RT-PCR

Bacteria grown on GC agar plates were suspended in 20 ml of GC broth to an OD_600 _of 0.1 and continuously cultured to mid-log phase (OD_600 _of ~ 0.6) at 37°C with shaking. The total RNA was isolated from the harvested bacteria as previously described [46] with an additional treatment with DNase I. RT-PCR was performed using one step RT-PCR Kit Ver. 1.1 (Takara Bio, Japan) with approximately 2 μg of total RNA according to the manufacturer's instructions. The products were visualized by electrophoresis in a 2 % agarose gel followed by ethidium bromide staining.

### Primer extension analysis

Fifty micrograms of total RNA and 5 pmol of biotin-labeled primer (ggt-ext-2) were hybridized in 20 μl of buffer (10 mM Tris-HCl, pH 8.0, 1 mM EDTA, 250 mM KCl). The hybridized RNA-DNA probe was treated with 35 units of AMV reverse transcriptase (RTase) XL (Takara Bio) in a reaction mixture (250 μM dNTPs, 1 × AMV reverse transcriptase XL buffer) at 37°C for 30 min. The ethanol-precipitated DNA product was dissolved in 20 μl of formamide dye (80 % formamide, 10 mM NaOH, 1 mM EDTA, 0.025 % bromophenol blue, 0.025 % xylene cyanol). Sequencing of the *ggt *and *ggh *genes with the ggt-ext-2 primer was performed by using *ΔTh *polymerase sequencing high -cycle- (TOYOBO, Japan). Aliquots of the reaction products were analyzed by electrophoresis on 8 % acrylamide-7 M urea gel followed by capillary blotting to Hybond-N+ (Amersham). The bands were visualized with Imaging high (TOYOBO) according to the manufacturer's protocol.

### SDS-PAGE and Western blotting

SDS-PAGE and Western blotting were performed as described previously [43] using 1 × 10^3^-fold diluted anti-GGT polyclonal rabbit antiserum [25] and 2 × 10^3^-fold diluted horseradish peroxidase-conjugated secondary antibody (Amersham)

## Abbreviations

GGT, γ-glutamyl transpeptidase; IS, insertional sequence; ORF, open reading frame; RTase, reverse transcriptase

## Authors' contributions

HT carried out all of the studies including molecular genetic studies, sequence determination, sequence analyses and drafting the manuscript. HW made a critical reading of the manuscript and final approval of the version to be published.

## Supplementary Material

Additional File 1 Alignment of the nucleotide sequences within the *ggt *and *ggh *genes of *N. meningitidis *H44/76 [DDBJ:AB089320]; *N. meningitidis *H119/90 [DDBJ: AB211221]; *N. gonorrhoeae *ATCC49226 [DDBJ:AB175023]; *N. gonorrhoeae *NIID103 [DDBJ:AB175025] and *N. gonorrhoeae *NIID106 [DDBJ:AB175029] strains, respectively. Sequence identity is represented as *, polymorphism within the sequences of the 5 strains is indicated by the appropriate letter, and the absence of a base is shown with a hyphen (-). Click here for file

## Figures and Tables

**Table 1:** Oligonucleotides used in this study

Primers for the sequencing of *ggt *and *ggh *genes, and RT-PCR (for ggt-9 and ggt-10)				
Oligonucleotide name	Position in sequence	Length (bp)	Sequence (5'-3')	Reference

ggt-3	*1265–1286	21	GACTGCTGATGACATTAGCGG	[49]
ggt-4	*3250–3228	22	GATTACTCACAATTTCCCCCTA	[49]
ggt-5	*1791–1811	20	CGATGCGTGCGACGCCGGAA	[25]
ggt-6	*2676–2654	23	ATAGCACATTGCCCGCCTTATCC	[25]
ggt-7	*2241–2262	22	CAAGATTTATCTGATTATCAAG	[25]
ggt-9	*2779–2800	21	GGGCAAACAGGTCGCCAATCG	[25]
ggt-10	*2089–2068	21	TGTAGCGGCACACCATTCGGC	[25]
ggt-18	*1554–1534	21	CGGTCAGTCCCGTTGCATGTT	[25]
Primers for RT-PCR				
ggt-29	*1452–1475	24	GGATGTCAAGTCATCCATGCCAAT	This study
ggt-20	*1682–1659	24	TGTCGTCTGCACCGCCACCATCGC	This study
ggt-31	*1878–1901	24	GGTACGCCTGCTATCCCTAAACTG	This study
ggt-22	*3079–3056	24	CGCACATCAGTCTTATAGCCCAAA	This study
Primer for primer extension				
primer-ext-2	*1492–1467	26	GTATTAACCTTACCTTGATTGGCATG (Biotin-labeled at the 5'-terminus)	This study

*Numbers of positions indicate the position from the 5'-nucleotide of the *ggt *locus in *N. meningitidis *strain H44/76 [DDBJ:AB175033].

**Table 2:** Mutation types found in the *ggh *genes of 11 *N. gonorrhoeae *strains

	Mutation type*			
	
Strains	I (+6 bp)	II (ochre)	III (Δ7 bp)	IV (+46 bp)
ATCC49226				
NIID102				
NIID104	-	+	+	+
NIID108				
NIID111				
NIID109 (48^th ^A to G)				
NIID103	+	+	+	+
NIID54	+	+	+	-
NIID106				
NIID107	-	+	+	-
NIID105 (213^th ^G to A)				

"+" or "-" denotes the presence or absence of the mutation, respectively.

*Mutation type corresponds to the categories in Figure 2B.

## References

[B1] Nassif X (1999). Interaction mechanisms of encapsulated meningococci with eucaryotic cells: what does this tell us about the crossing of the blood-brain barrier by Neisseria meningitidis?. Curr Opin Microbiol.

[B2] Suzuki H, Hashimoto W, Kumagai H (1999). Glutathione metabolism in Escherichia coli. J Mol Catal B.

[B3] Chevalier C, Thiberge JM, Ferrero RL, Labigne A (1999). Essential role of Helicobacter pylori γ glutamyltranspeptidase for the colonization of the gastric mucosa of mice. Mol Microbiol.

[B4] McGovern KJ, Blanchard TG, Gutierrez JA, Czinn SJ, Krakowka S, Youngman P (2001). γ Glutamyltransferase is a Helicobacter pylori virulence factor but is not essential for colonization. Infect Immun.

[B5] D'Amato RF, Eriquez LA, Tomfohrde KM, Singerman E (1978). Rapid identification of Neisseria gonorrhoeae and Neisseria meningitidis by using enzymatic profiles. J Clin Microbiol.

[B6] Brown JD, Thomas KR (1985). Rapid enzyme system for the identification of pathogenic Neisseria spp. J Clin Microbiol.

[B7] Janda WM, Ulanday MG, Bohnhoff M, LeBeau LJ (1985). Evaluation of the RIM-N, Gonochek II, and Phadebact systems for the identification of pathogenic Neisseria spp. and Branhamella catarrhalis. J Clin Microbiol.

[B8] Philip A, Garton GC (1985). Comparative evaluation of five commercial systems for the rapid identification of pathogenic Neisseria species. J Clin Microbiol.

[B9] Welborn PP, Uyeda CT, Ellison-Birang N (1984). Evaluation of Gonochek-II as a rapid identification system for pathogenic Neisseria species. J Clin Microbiol.

[B10] Ramos-Onsins S, Aguade M (1998). Molecular evolution of the Cecropin multigene family in Drosophila. functional genes vs. pseudogenes. Genetics.

[B11] Loguercio LL, Wilkins TA (1998). Structural analysis of a hmg-coA-reductase pseudogene: insights into evolutionary processes affecting the hmgr gene family in allotetraploid cotton (Gossypium hirsutum L.). Curr Genet.

[B12] Mighell AJ, Smith NR, Robinson PA, Markham AF (2000). Vertebrate pseudogenes. FEBS Lett.

[B13] Lawrence JG, Hendrix RW, Casjens S (2001). Where are the pseudogenes in bacterial genomes?. Trends Microbiol.

[B14] Hall BG, Yokoyama S, Calhoun DH (1983). Role of cryptic genes in microbial evolution. Mol Biol Evol.

[B15] Schnetz K, Rak B (1988). Regulation of the bgl operon of Escherichia coli by transcriptional antitermination. EMBO J.

[B16] Parker LL, Hall BG (1990). Characterization and nucleotide sequence of the cryptic cel operon of Escherichia coli K12. Genetics.

[B17] Al Mamun AA, Tominaga A, Enomoto M (1996). Detection and characterization of the flagellar master operon in the four Shigella subgroups. J Bacteriol.

[B18] Al Mamun AA, Tominaga A, Enomoto M (1997). Cloning and characterization of the region III flagellar operons of the four Shigella subgroups: genetic defects that cause loss of flagella of Shigella boydii and Shigella sonnei. J Bacteriol.

[B19] Tominaga A, Mahmoud MA, Mukaihara T, Enomoto M (1994). Molecular characterization of intact, but cryptic, flagellin genes in the genus Shigella. Mol Microbiol.

[B20] Andersson JO, Andersson SG (2001). Pseudogenes, junk DNA, and the dynamics of Rickettsia genomes. Mol Biol Evol.

[B21] Tettelin H, Saunders NJ, Heidelberg J, Jeffries AC, Nelson KE, Eisen JA, Ketchum KA, Hood DW, Peden JF, Dodson RJ, Nelson WC, Gwinn ML, DeBoy R, Peterson JD, Hickey EK, Haft DH, Salzberg SL, White O, Fleischmann RD, Dougherty BA, Mason T, Ciecko A, Parksey DS, Blair E, Cittone H, Clark EB, Cotton MD, Utterback TR, Khouri H, Qin H, Vamathevan J, Gill J, Scarlato V, Masignani V, Pizza M, Grandi G, Sun L, Smith HO, Fraser CM, Moxon ER, Rappuoli R, Venter JC (2000). Complete genome sequence of Neisseria meningitidis serogroup B strain MC58. Science.

[B22] Andersson JO, Andersson SG (1999). Genome degradation is an ongoing process in Rickettsia. Mol Biol Evol.

[B23] Zhu P, Morelli G, Achtman M (1999). The opcA and ψopcB regions in Neisseria: genes, pseudogenes, deletions, insertion elements and DNA islands. Mol Microbiol.

[B24] Feavers IM, Maiden MC (1998). A gonococcal porA pseudogene: implications for understanding the evolution and pathogenicity of Neisseria gonorrhoeae. Mol Microbiol.

[B25] Takahashi H, Watanabe H (2004). Post-translational processing of Neisseria meningitidis γ-glutamyl aminopeptidase and its association with inner membrane facing to the cytoplasmic space. FEMS Microbiol Lett.

[B26] Reyes A, Mezzina M, Gadaleta G (2002). Human mitochondrial transcription factor A (mtTFA): gene structure and characterization of related pseudogenes. Gene.

[B27] Asakura N, Nakamura C, Ishii T, Kasai Y, Yoshida S (2002). A transcriptionally active maize MuDR-like transposable element in rice and its relatives. Mol Genet Genomics.

[B28] Moreau-Aubry A, Le Guiner S, Labarriere N, Gesnel MC, Jotereau F, Breathnach R (2000). A processed pseudogene codes for a new antigen recognized by a CD8(+) T cell clone on melanoma. J Exp Med.

[B29] Hirotsune S, Yoshida N, Chen A, Garrett L, Sugiyama F, Takahashi S, Yagami K, Wynshaw-Boris A, Yoshiki A (2003). An expressed pseudogene regulates the messenger-RNA stability of its homologous coding gene. Nature.

[B30] Lees-Murdock DJ, McLoughlin GA, McDaid JR, Quinn LM, O'Doherty A, Hiripi L, Hack CJ, Walsh CP (2004). Identification of 11 pseudogenes in the DNA methyltransferase gene family in rodents and humans and implications for the functional loci. Genomics.

[B31] Korneev SA, Park JH, O'Shea M (1999). Neuronal expression of neural nitric oxide synthase (nNOS) protein is suppressed by an antisense RNA transcribed from an NOS pseudogene. J Neurosci.

[B32] Dehio C, Gray-Owen SD, Meyer TF (1998). The role of neisserial Opa proteins in interactions with host cells. Trends Microbiol.

[B33] Nassif X, Pujol C, Morand P, Eugene E (1999). Interactions of pathogenic Neisseria with host cells. Is it possible to assemble the puzzle?. Mol Microbiol.

[B34] Baranov PV, Hammer AW, Zhou J, Gesteland RF, Atkins JF (2005). Transcriptional slippage in bacteria: distribution in sequenced genomes and utilization in IS element gene expression. Genome Biol.

[B35] Mira A, Pushker R (2005). The Silencing of Pseudogenes. Mol Biol Evol.

[B36] Hart CA, Rogers TR (1993). Meningococcal disease. J Med Microbiol.

[B37] van Deuren M, Brandtzaeg P, van der Meer JW (2000). Update on meningococcal disease with emphasis on pathogenesis and clinical management. Clin Microbiol Rev.

[B38] Tunkel AR, Scheld WM (1993). Pathogenesis and pathophysiology of bacterial meningitis. Clin Microbiol Rev.

[B39] Takahashi H, Hirose K, Watanabe H (2004). Necessity of meningococcal γ-glutamyl aminopeptidase for the Neisseria meningitidis growth in rat cerebrospinal fluid (CSF) and CSF mimicking medium. J Bacteriol.

[B40] Takahashi H, Kuroki T, Watanabe Y, Dejsirilert S, Saengsuk L, Yamai S, Watanabe H (2004). Reliability of the detection of meningococcal γ-glutamyl transpeptidase as an identification marker for Neisseria meningitidis. Microbiol Immunol.

[B41] Takahashi H, Kuroki T, Watanabe Y, Tanaka H, Inouye H, Yamai S, Watanabe H (2004). Characterization of Neisseria meningitidis isolates collected from 1974 to 2003 in Japan by multilocus sequence typing. J Med Microbiol.

[B42] Takahashi H, Watanabe H (2002). A broad-host-range vector of incompatibility group Q can work as a plasmid vector in Neisseria meningitidis: a new genetical tool. Microbiology.

[B43] phylogenic tree prediction. http://www.genebee.msu.ru/services/phtree_reduced.html.

[B44] Huson DH (1998). SplitsTree: analyzing and visualizing evolutionary data. Bioinformatics.

[B45] Takahashi H, Inada T, Postma P, Aiba H (1998). CRP down-regulates adenylate cyclase activity by reducing the level of phosphorylated IIA(Glc), the glucose-specific phosphotransferase protein, in Escherichia coli. Mol Gen Genet.

[B46] Neisseria gonorrhoeae genome sequencing strain FA1090. http://www.genome.ou.edu/gono.html.

[B47] The Sanger Institute: Neisseria lactamica. http://www.sanger.ac.uk/Projects/N_lactamica/.

[B48] Takahashi H, Tanaka H, Inouye H, Kuroki T, Watanabe Y, Yamai S, Watanabe H (2002). Isolation from a healthy carrier and characterization of a Neisseria meningitidis strain that is deficient in γ-glutamyl aminopeptidase activity. J Clin Microbiol.

