# Supplementary material for: A gonococcal homologue of meningococcal γ-glutamyl transpeptidase gene is a new type of bacterial pseudogene that is transcriptionally active but phenotypically silent
Source: BMC Microbiol. 2005 Oct 4;5:56. doi: 10.1186/1471-2180-5-56 (PMC1262726; doi:10.1186/1471-2180-5-56)
Supplement: Additional File 1 — Alignment of the nucleotide sequences within the ggt and ggh genes of N. meningitidis H44/76 [DDBJ:AB089320]; N. meningitidis H119/90 [DDBJ: AB211221]; N. gonorrhoeae ATCC49226 [DDBJ:AB175023]; N. gonorrhoeae NIID103 [DDBJ:AB175025] and N. gonorrhoeae NIID106 [DDBJ:AB175029] strains, respectively. Sequence identity is represented as *, polymorphism within the sequences of the 5 strains is indicated by the appropriate letter, and the absence of a base is shown with a hyphen (-). [file 1471-2180-5-56-S1.pdf]

```
H44/76          1       ATGCCTTGTATGAATCATCAATCAAACTCA------GGCGAAGGAGTGCTTGTGGCTAAA    54
H114/90         1       ATGCCTTGTATGAATCATCAATCAAACTCA------GGCGAAGGAGTGCTTGTGGCTAAA    54
ATCC49226       1       ATGCTTTGTATGAATTATCAATCAAACTCA------GGCGAAGGAGTGCTTGTAGCTAAA    54
NIID54          1       ATGCTTTGTATGAATTATCAATCAAACTCAAACTCAGGCGAAGGAGTGCTTGTGGCTAAA    60
NIID103         1       ATGCTTTGTATGAATTATCAATCAAACTCAAACTCAGGCGAAGGAGTGCTTGTGGCTAAA    60
NIID106         1       ATGCTTTGTATGAATTATCAATCAAACTCA------GGCGAAGGAGTGCTTGTGGCTAAA    54
                        **** ********** **************      ***************** ******

H44/76          55      ACATATTTATTGACTGCATTGATAATGTCTATGACAATCTCTGGATGTCAAGTCATCCAT    114
H114/90         55      ACATATTTATTGACTGCATTGATAATGTCTATGACAATCTCTGGATGTCAAGTCATCCAT    114
ATCC49226       55      ACATATTTATTGACTGCATTGATAATGTCTATGGTAATCTCCGGATGTCAAGTCATCCAT    114
NIID54          61      ACATATTTATTGACTGCATTGATAATGTCTATGGTAATCTCCGGATGTCAAGTCATCCAT    120
NIID103         61      ACATATTTATTGACTGCATTGATAATGTCTATGGTAATCTCCGGATGTCAAGTCATCCAT    120
NIID106         55      ACATATTTATTGACTGCATTGATAATGTCTATGGTAATCTCCGGATGTCAAGTCATCCAT    114
                        *********************************  ****** ******************

H44/76          115     GCCAATCAAGGTAAGGTTAATACTCATTCTGCTGTCATCACAGGTGCAGACGCTCACACG    174
H114/90         115     GCCAATCAAGGTAAGGTTAATACTCATTCTGCTGTCATCACAGGTGCAGACGCTCACACG    174
ATCC49226       115     GCCAATCAAGGTAAGGTTAATACTAATTCTGCTGTCATCGCAGGTGCAGACGCTCACACG    174
NIID54          121     GCCAATCAAGGTAAGGTTAATACTAATTCTGCTGTCATCGCAGGTGCAGACGCTCACACG    180
NIID103         121     GCCAATCAAGGTAAGGTTAATACTAATTCTGCTGTCATCGCAGGTGCAGACGCTCACACG    180
NIID106         115     GCCAATCAAGGTAAGGTTAATACTAATTCTGCTGTCATCGCAGGTGCAGACGCTCACACG    174
                        ************************ ************** ********************

H44/76          175     CCTGAACATGCAACGGGACTGACCGAACAAAAGCAGGTGATTGCAAGTGATTTTATGGTA    234
H114/90         175     CCTGAACATGCAACGGGACTGATCGAACAAAAGCAGGTGATTGCAAGTGATTTTATGGTA    234
ATCC49226       175     CCTGAACATGTAACGGGACTGACCGAACAAAAGCAGGTGATTGCAAGTGATTTTATAGTA    234
NIID54          181     CCTGAACATGTAACGGGACTGACCGAACAAAAGCAGGTGATTGCAAGTGATTTTATAGTA    240
NIID103         181     CCTGAACATGTAACGGGACTGACCGAACAAAAGCAGGTGATTGCAAGTGATTTTATAGTA    240
NIID106         175     CCTGAACATGTAACGGGACTGACCGAACAAAAGCAGGTGATTGCAAGTGATTTTATAGTA    234
                        ********** *********** ********************************* ***

H44/76          235     GCGTCAGCCAATCCATTAGCAACACAAGCTGGCTATGATATCTTAAAGCAAGGCGGTAGC    294
H114/90         235     GCGTCAGCCAATCCATTAGCAACACAAGCTGGCTATGATATCTTAAAGCAAGGCGGTAGC    294
ATCC49226       235     GCGTCAGCCAATCCATTAGCAACACAAGCTGGCTATGATATCTTAAAGCAAGGCGGTAGC    294
NIID54          241     GCGTCAGCCAATCCATTAGCAACACAAGCTGGCTATGATATCTTAAAGCAAGGCGGTAGC    300
NIID103         241     GCGTCAGCCAATCCATTAGCAACACAAGCTGGCTATGATATCTTAAAGCAAGGCGGTAGC    300
NIID106         235     GCGTCAGCCAATCCATTAGCAACACAAGCTGGCTATGATATCTTAAAGCAAGGCGGTAGC    294
                        ************************************************************

H44/76          295     GCTGCAGATGCGATGGTGGCGGTGCAGACGACACTAAGCTTGGTAGAGCCACAGTCGTCA    354
H114/90         295     GCTGCAGATGCGATGGTGGCGGTGCAGACGACACTAAGCTTGGTAGAGCCACAGTCGTCA    354
ATCC49226       295     GCTGCAGATGCGATGGTGGCGGTGCAGACGACACTAAGCTTGGTAGAGCCACAGTCGTCA    354
NIID54          295     GCTGCAGATGCGATGGTGGCGGTGCAGACGACACTAAGCTTGGTAGAGCCACAGTCGTCA    360
NIID103         295     GCTGCAGATGCGATGGTGGCGGTGCAGACGACACTAAGCTTGGTAGAGCCACAGTCGTCA    360
NIID106         295     GCTGCAGATGCGATGGTGGCGGTGCAGACGACACTAAGCTTGGTAGAGCCACAGTCGTCA    354
                        ************************************************************

H44/76          355     GGCTTGGGCGGTGGTGCATTTGTGTTGTATTGGGATAATACCGCCAAAACATTGACCACA    414
H114/90         355     GGCTTGGGCGGTGGTGCATTTGTGTTGTATTGGGATAATACCGCCAAAACATTGACCACA    414
ATCC49226       355     GGCTTGGGCGGTGGTGCATTTGTGTTGTATTGGGACAATACCGCCAAAACATTGACCACA    414
NIID54          361     GGCTTGGGCGGTGGTGCATTTGTGTTGTATTGGGACAATACCGCCAAAACATTGACCACA    420
NIID103         361     GGCTTGGGCGGTGGTGCATTTGTGTTGTATTGGGACAATACCGCCAAAACATTGACCACA    420
NIID106         355     GGCTTGGGCGGTGGTGCATTTGTGTTGTATTGGGACAATACCGCCAAAACATTGACCACA    414
                        *********************************** ************************

H44/76          361     TTTGATGGGCGTGAGACGGCACCGATGCGTGCGACGCCGGAATTATTTTTGGATAAAGAT    474
H114/90         361     TTTGATGGGCGTGAGACGGCACCGATGCGTGCGACGCCGGAATTATTTTTGGATAAAGAT    474
ATCC49226       361     TTTGATGGGCGTGAGACGGCACCGATGCGTGCGACGCCAGAATTATTTTTGGATAAAGAT    474
NIID54          421     TTTGATGGGCGTGAGACGGCACCGATGCGTGCGACGCCAGAATTATTTTTGGATAAAGAT    480
NIID103         421     TTTGATGGGCGTGAGACGGCACCGATGCGTGCGACGCCAGAATTATTTTTGGATAAAGAT    480
NIID106         361     TTTGATGGGCGTGAGACGGCACCGATGCGTGCGACGCCAGAATTATTTTTGGATAAAGAT    474
                        ************************************** *********************

H44/76          475     GGTCAACCATTGAAATTTATGGAAGCGGTGGTCGGTGGTCGCTCGGTGGGTACGCCTGCT    534
H114/90         475     GGTCAACCATTGAAATTTATGGAAGCGGTGGTCGGTGGTCGCTCGGTGGGTACGCCTGCT    534
ATCC49226       475     GGTTAACCATTGAAATTTATGGAAGCGGTGGTCG-------CTCGGTAGGTACGCCTGCT    527
NIID54          481     GGTTAACCATTGAAATTTATGGAAGCGGTGGTCG-------CTCGGTAGGTACGCCTGCT    533
NIID103         481     GGTTAACCATTGAAATTTATGGAAGCGGTGGTCG-------CTCGGTAGGTACGCCTGCT    533
NIID106         475     GGTTAACCATTGAAATTTATGGAAGCGGTGGTCG-------CTCGGTAGGTACGCCTGCT    527
                        *** ******************************       ****** ************

H44/76          535     ATCCCTAAACTGATGGAGACAATACATCAGCGATACGGTGTATTGCCTTGGGGAAAATTA    594
H114/90         535     ATCCCTAAACTGATGGAGACAATACATCAGCGATACGGTGTATTGCCTTGGGGAAAATTA    594
ATCC49226       528     ATCCCTAAACTGACGGAGACAATACATCAGCGATACGGTGTATTGCCTTGGGGAAAATTA    587
NIID54          534     ATCCCTAAACTGACGGAGACAATACATCAGCGATACGGTGTATTGCCTTGGGGAAAATTA    593
NIID103         534     ATCCCTAAACTGACGGAGACAATACATCAGCGATACGGTGTATTGCCTTGGGGAAAATTA    593
NIID106         528     ATCCCTAAACTGACGGAGACAATACATCAGCGATACGGTGTATTGCCTTGGGGAAAATTA    587
                        ************* **********************************************

NIID103         595     TTTGATACGCCGATCCACTTGGCAAAACAAGGCTTTGAGGTGTCGCCAAGGCTTGCCATC    654
ATCC49226       595     TTTGATACGCCGATCCACTTGGCAAAACAAGGCTTTGAGGTGTCGCCAAGGCTTGCCATC    654
NIID54          588     TTTGATACGCCGATCCACTTGGCAAAACAAGGCTTTGAGGTGTCGCCAAGGCTTGCCATC    647
NIID106         594     TTTGATACGCCGATCCACTTGGCAAAACAAGGCTTTGAGGTGTCGCCAAGGCTTGCCATC    653
H114/90         594     TTTGATACACCGATCCGCTTGGCAAAACAAGGCTTTGAGGTGTCGCCAAGGCTTGCCATC    653
H44/76          588     TTTGATACACCGATCCGCTTGGCAAAACAAGGCTTTGAGGTGTCGCCAAGGCTTGCCATC    647
                        ******** ******* *******************************************

H44/76          655     TCGGTTGAGCAAAATCAGCAGCATTTGGCACGCTATCCAAAAACAGCCGCTTATTTTTTG    714
H114/90         655     TCGGTTGAGCAAAATCAGCAGCATTTGGCACGCTATCCAAAAACAGCCGCTTATTTTTTG    714
ATCC49226       648     TCGGTTGAGCAAAATCAGCAGCATTTGGCACGCTATCCAAAAACAGCCGCTTATTTTTTG    707
NIID54          654     TCGGTTGAGCAAAATCAGCAGCATTTGGCACGCTATCCAAAAACAGCCGCTTATTTTTTG    713
NIID103         654     TCGGTTGAGCAAAATCAGCAGCATTTGGCACGCTATCCAAAAACAGCCGCTTATTTTTTG    713
NIID106         648     TCGGTTGAGCAAAATCAGCAGCATTTGGCACGCTATCCAAAAACAGCCGCTTATTTTTTG    707
                        ************************************************************

H44/76          715     CCGAATGGTGTGCCGCTACAAGCAGGCAGCTTGCTGAAAAATTTAGAATTTGCTGACAGT    774
H114/90         715     CCGAATGGTGTGCCGCTACAAGCAGGCAGCTTGCTGAAAAATTTAGAATTTGCTGACAGT    774
ATCC49226       708     CCGAATGGTGTGCCGCTACAAGCAGGCAGCTTGCTGAAAAATTTAGAATTTGCTGACAGT    767
NIID54          714     CCGAATGGTGTGCCGCTACAAGCAGGCAGCTTGCTGAAAAATTTAGAATTTGCTGACAGT    773
NIID103         714     CCGAATGGTGTGCCGCTACAAGCAGGCAGCTTGCTGAAAAATTTAGAATTTGCTGACAGT    773
NIID106         708     CCGAATGGTGTGCCGCTACAAGCAGGCAGCTTGCTGAAAAATTTAGAATTTGCTGACAGT    767
                        ************************************************************

H44/76          775     GTTCAGGCGTTAGCAGCTCAAGGTGCAAAAGCTCTGCATACTGGTAAATATGCCCAAAAT    834
H114/90         775     GTTCAGGCGTTAGCAGCTCAAGGTGCAAAAGCTCTGCATACTGGTAAATATGCCCAAAAT    834
ATCC49226       768     GTTCAGGCGTTAGCAGCTCAAGGTGCAAAAGCTCTGCATACTGGTAAATATGCCCAAAAT    827
NIID54          774     GTTCAGGCGTTAGCAGCTCAAGGTGCAAAAGCTCTGCATACTGGTAAATATGCCCAAAAT    833
NIID103         774     GTTCAGGCGTTAGCAGCTCAAGGTGCAAAAGCTCTGCATACTGGTAAATATGCCCAAAAT    833
NIID106         768     GTTCAGGCGTTAGCAGCTCAAGGTGCAAAAGCTCTGCATACTGGTAAATATGCCCAAAAT    827
                        ************************************************************

H44/76          835     ATCGTTTCAGTTGTCCAAAATGCTAAGGATAACCCCGGTCAATTATCCTTACAAGATTTA    894
H114/90         835     ATCGTTTCAGTTGTCCAAAATGCTAAGGATAACCCCGGTCAATTATCCTTACAAGATTTA    894
ATCC49226       828     ATTGTTTCAGTTGTCCAAAATGCTAAGGATAACCCCGGTCAATTATCCTTGCAAAATTTA    887
NIID54          834     ATTGTTTCAGTTGTCCAAAATGCTAAGGATAACCCCGGTCAATTATCCTTGCAAAATTTA    893
NIID103         834     ATTGTTTCAGTTGTCCAAAATGCTAAGGATAACCCCGGTCAATTATCCTTGCAAAATTTA    893
NIID106         828     ATTGTTTCAGTTGTCCAAAATGCTAAGGATAACCCCGGTCAATTATCCTTGCAAAATTTA    887
                        ** *********************************************** *** *****

H44/76          895     TCTGATTATCAAGTGGTGGAACGCCCGCCTGTTTGTGTGACTTATCGTATTTATGAAGTA    954
H114/90         895     TCTGATTATCAAGTGGTGGAACGCCCGCCTGTTTGTGTGACTTATCGTATTTATGAAGTA    954
ATCC49226       888     TCTGATTATCAAGTGGTGGAACGCCCGCCTGTTTGTGTGACTTATCGTATTTATGAAGTA    947
NIID54          894     TCTGATTATCAAGTGGTGGAACGCCCGCCTGTTTGTGTGACTTATCGTATTTATGAAGTA    953
NIID103         894     TCTGATTATCAAGTGGTGGAACGCCCGCCTGTTTGTGTGACTTATCGTATTTATGAAGTA    953
NIID106         888     TCTGATTATCAAGTGGTGGAACGCCCGCCTGTTTGTGTGACTTATCGTATTTATGAAGTA    947
                        ************************************************************

H44/76          955     TGCGGTATGGGTGCACCAAGCTCAGGTGGGATTGCTGTGGGTCAGATTTTGGGGATTTTA    1014
H114/90         955     TGCGGTATGGGTGCACCAAGCTCAGGTGGGATTGCTGTGGGTCAAATTTTGGGGATTTTA    1014
ATCC49226       948     TGCGGTATGGGTGCACCAAGCTCAGGTGGGATTGCTGTGGGTCAGATTTTGGGGATTTTA    1007
NIID54          954     TGCGGTATGGGTGCACCAAGCTCAGGTGGGATTGCTGTGGGTCAGATTTTGGGGATTTTA    1013
NIID103         954     TGCGGTATGGGTGCACCAAGCTCAGGTGGGATTGCTGTGGGTCAGATTTTGGGGATTTTA    1013
NIID106         948     TGCGGTATGGGTGCACCAAGCTCAGGTGGGATTGCTGTGGGTCAGATTTTGGGGATTTTA    1007
                        ******************************************** ***************

H44/76          1015    AATGAATTTTCACCCAATCAGGTGGGGTATGATGCTGAAGGTTTACGTCTCTTGGGCGAC    1074
H114/90         1015    AATGAATTTTCACCCAATCAGGTGGGGTATGATGCTGAAGGTTTACGTCTCTTGGGCGAC    1074
ATCC49226       1008    AATGAATTTTCACCCAATCGGGTGGGGTATGATGCTGAAGGTTTGCGTCTCTTGGGCGAT    1067
NIID54          1014    AATGAATTTTCACCCAATCGGGTGGGGTATGATGCTGAAGGTTTGCGTCTCTTGGGCGAT    1073
NIID103         1014    AATGAATTTTCACCCAATCGGGTGGGGTATGATGCTGAAGGTTTGCGTCTCTTGGGCGAT    1073
NIID106         1008    AATGAATTTTCACCCAATCGGGTGGGGTATGATGCTGAAGGTTTGCGTCTCTTGGGCGAT    1067
                        ******************* ************************ ************** 

H44/76          1075    GCTTCTAGGCTTGCGTTTGCGGATCGTGATGTATAT------------------------    1100
H114/90         1075    GCTTCTAGGCTTGCGTTTGCGGATCGTGATGTATAT------------------------    1100
ATCC49226       1068    GCTTCTAGGCTTGCGTTTGCGGATCGTGATGTATATCTTGGGCGATGCTTCTAGGCTTGC    1127
NIID54          1074    GCTTCTAGGCTTGCGTTTGCGGATCGTGATGTATAT------------------------    1109
NIID103         1074    GCTTCTAGGCTTGCGTTTGCGGATCGTGATGTATATCTTGGGCGATGCTTCTAGGCTTGC    1133
NIID106         1068    GCTTCTAGGCTTGCGTTTGCGGATCGTGATGTATAT------------------------    1103
                        ************************************

H44/76          1101    ----------------------TTGGGCGACCCTGATTTTGTACCAGTACCCATTCGCCA    1138
H114/90         1101    ----------------------TTGGGCGACCCTGATTTTGTACCAGTACCCATTCGCCA    1138
ATCC49226       1128    GTTTGCGGATCGTGATGTATATTTGGGCGACCCTGATTTTGTACCAGTACCCATTCGCCA    1187
NIID54          1110    ----------------------TTGGGCGACCCTGATTTTGTACCAGTACCCATTCGCCA    1147
NIID103         1134    GTTTGCGGATCGTGATGTATATTTGGGCGACCCTGATTTTGTACCAGTACCCATTCGCCA    1193
NIID106         1103    ----------------------TTGGGCGACCCTGATTTTGTACCAGTACCCATTCGCCA    1141
                                              **************************************

H44/76          1139    GTTGATTTCTAAAGACTACCTAAAACATCGCAGCCAGCTGCTTGAGCAGTCGGATAAGGC    1198
H114/90         1139    GTTGATTTCTAAAGACTACCTAAAACATCGCAGCCAGCTGCTTGAGCAGTCGGATAAGGC    1198
ATCC49226       1188    GTTGATTTCTAAAGACTACCTAAAACATCGCAGCCAGCTGCTTGAGCAGTCGGATAAGGC    1247
NIID54          1148    GTTGATTTCTAAAGACTACCTAAAACATCGCAGCCAGCTGCTTGAGCAGTCGGATAAGGC    1207
NIID103         1194    GTTGATTTCTAAAGACTACCTAAAACATCGCAGCCAGCTGCTTGAGCAGTCGGATAAGGC    1253
NIID106         1142    GTTGATTTCTAAAGACTACCTAAAACATCGCAGCCAGCTGCTTGAGCAGTCGGATAAGGC    1201
                        ************************************************************

H44/76          1199    ATTGCCTAGCGTATCTGCAGGCGATTTTATTCATGAGTGGGTGTCCTCACAAGCGATTGA    1258
H114/90         1199    ATTGCCTAGCGTATCTGCAGGCGATTTTATTCATGAGTGGGTGTCCTCACAAGCGATTGA    1258
ATCC49226       1248    ATTGCCTAGCGTATCTGCAGGCGATTTTATTCATGAGTGGGCGTCCTCACAAGCGATTGA    1307
NIID54          1208    ATTGCCTAGCGTATCTGCAGGCGATTTTATTCATGAGTGGGCGTCCTCACAAGCGATTGA    1267
NIID103         1254    ATTGCCTAGCGTATCTGCAGGCGATTTTATTCATGAGTGGGCGTCCTCACAAGCGATTGA    1313
NIID106         1202    ATTGCCTAGCGTATCTGCAGGCGATTTTATTCATGAGTGGGCGTCCTCACAAGCGATTGA    1261
                        ***************************************** ******************

H44/76          1259    GTTACCTTCTACCAGTCATATATCAATTGTGGATAAGGCGGGCAATGTGCTATCAATGAC    1318
H114/90         1259    GTTACCTTCTACCAGTCATATATCAATTGTGGATAAGGCGGGCAATGTGCTATCAATGAC    1318
ATCC49226       1308    GTTACCTTCTACCAGTCATATATCAATTGTGGATAAGGCGGGCAATGTGCTATCAATGAC    1367
NIID54          1268    GTTACCTTCTACCAGTCATATATCAATTGTGGATAAGGCGGGCAATGTGCTATCAATGAC    1327
NIID103         1314    GTTACCTTCTACCAGTCATATATCAATTGTGGATAAGGCGGGCAATGTGCTATCAATGAC    1373
NIID106         1262    GTTACCTTCTACCAGTCATATATCAATTGTGGATAAGGCGGGCAATGTGCTATCAATGAC    1321
                        ************************************************************

H44/76          1319    GACTTCCATTGAAAATGCGTTTGGCTCAACTTTGATGGCAAATGGCTACCTGCTGAATAA    1378
H114/90         1319    GACTTCCATTGAAAATGCGTTTGGCTCAACTTTGATGGCAAATGGCTACCTGCTGAATAA    1378
ATCC49226       1368    GACTTCCATTGAAAATGCGTTTGGCTCAACTTTGATGGCAAATGGCTACCTGCTGAATAA    1427
NIID54          1328    GACTTCCATTGAAAATGCGTTTGGCTCAACTTTGATGGCAAATGGCTACCTGCTGAATAA    1387
NIID103         1374    GACTTCCATTGAAAATGCGTTTGGCTCAACTTTGATGGCAAATGGCTACCTGCTGAATAA    1433
NIID106         1322    GACTTCCATTGAAAATGCGTTTGGCTCAACTTTGATGGCAAATGGCTACCTGCTGAATAA    1381
                        ************************************************************

H44/76          1379    TGAGCTGACAGACTTTAGTTTTGAGCCAATAAAGCAGGGCAAACAGGTCGCCAATCGTGT    1438
H114/90         1379    TGAGCTGACAGACTTTAGTTTTGAGCCAATAAAGCAGGGCAAACAGGTCGCCAATCGTGT    1438
ATCC49226       1428    TGAGCTGACAGACTTTAGTTTTGAGCCAATAAAGCAGGGCAAACAGGTCGCCAATCGTGT    1487
NIID54          1388    TGAGCTGACAGACTTTAGTTTTGAGCCAATAAAGCAGGGCAAACAGGTCGCCAATCGTGT    1447
NIID103         1434    TGAGCTGACAGACTTTAGTTTTGAGCCAATAAAGCAGGGCAAACAGGTCGCCAATCGTGT    1493
NIID106         1382    TGAGCTGACAGACTTTAGTTTTGAGCCAATAAAGCAGGGCAAACAGGTCGCCAATCGTGT    1441
                        ************************************************************

H44/76          1439    CGAGCCCGGCAAGAGACCACGCTCCTCAATGGCACCAACCATCGTATTTAAAGCAGGCAA    1498
H114/90         1439    CGAGCCCAGCAAGAGACCACGCTCCTCAATGGCACCAACCATCGTATTTAAAGCAGGCAA    1498
ATCC49226       1488    CGAGCCTGGCAAGAGACCACGCTCCTCAATGGCACCAACCATCGTATTTAAAGCAGGCAA    1547
NIID54          1448    CGAGCCTGGCAAGAGACCACGCTCCTCAATGGCACCAACCATCGTATTTAAAGCAGGCAA    1507
NIID103         1494    CGAGCCTGGCAAGAGACCACGCTCCTCAATGGCACCAACCATCGTATTTAAAGCAGGCAA    1553
NIID106         1442    CGAGCCTGGCAAGAGACCACGCTCCTCAATGACACCAACCATCGTATTTAAAGCAGGCAA    1501
                        ******  *********************** ****************************

H44/76          1499    GCCTTATATGGCAATCGGCTCCCCAGGTGGTAGCCGTATCATTGGCTATGTCGCTAAGAC    1558
H114/90         1499    GCCTTATATGGCAATCGGCTCCCCAGGTGGTAGCCGTATCATTGGCTATGTCGCTAAGAC    1558
ATCC49226       1548    GCCTTATATGGCAATCGGCTCTCCAGGTGGTAGCCGTATCATTGGCTATGTCGCTAAGAC    1607
NIID54          1508    GCCTTATATGGCAATCGGCTCTCCAGGTGGTAGCCGTATCATTGGCTATGTCGCTAAGAC    1567
NIID103         1554    GCCTTATATGGCAATCGGCTCTCCAGGTGGTAGCCGTATCATTGGCTATGTCGCTAAGAC    1613
NIID106         1502    GCCTTATATGGCAATCGGCTCTCCAGGTGGTAGCCGTATCATTGGCTATGTCGCTAAGAC    1561
                        ********************* **************************************

H44/76          1559    GATTGTGGCACATAGTGACTGGAATATGGATATCCAAAATGCCATCAGTGCACCCAATTT    1618
H114/90         1559    GATTGTGGCACATAGCGACTGGAATATGGATATCCAAGATGCCATCAGTGCACCCAATTT    1618
ATCC49226       1608    GATTGTGGCACATAGTGACTGGAATATGGATATCCAAGATGCCATCAGTGCACCCAATTT    1667
NIID54          1568    GATTGTGGCACATAGTGACTGGAATATGGATATCCAAGATGCCATCAGTGCACCCAATTT    1627
NIID103         1614    GATTGTGGCACATAGTGACTGGAATATGGATATCCAAGATGCCATCAGTGCACCCAATTT    1673
NIID106         1562    GATTGTGGCACATAGTGACTGGAATATGGATATCCAAGATGCCATCAGTGCACCCAATTT    1621
                        *************** ******************** ***********************

H44/76          1619    ATTAAATCGCTTTGGTAGCTATGAACTAGAAACGGGTACAACCGCCGTCCAGTGGCAACA    1678
H114/90         1619    ATTAAATCGCTTTGGTAGCTATGAACTAGAAACGGGTACAACCGCCCTCCAGTGGCAACA    1678
ATCC49226       1668    ATTAAATCGCTTTGGTAGCTATGAACTAGAAACGGGTACAACCGCCGTCCAGTGGCAACA    1727
NIID54          1628    ATTAAATCGCTTTGGTAGCTATGAACTAGAAACGGGTACAACCGCCGTCCAGTGGCAACA    1687
NIID103         1674    ATTAAATCGCTTTGGTAGCTATGAACTAGAAACGGGTACAACCGCCGTCCAGTGGCAACA    1733
NIID106         1622    ATTAAATCGCTTTGGTAGCTATGAACTAGAAACGGGTACAACCGCCGTCCAGTGGCAACA    1681
                        ********************************************** *************

H44/76          1679    AGCATTAAATGATTTGGGCTATAAGACTGATGTGCGTGAGCTAAATTCAGGTGTTCAAGC    1738
H114/90         1679    AGCATTAAATGATTTGGGCTATAAGACTGACGTGCGTGAGCTAAATTCAGGTGTTCAAGC    1738
ATCC49226       1728    AACATTAAATGATTTGGGCTATAAGACTGATGTGCGTGAGCTAAATTCAGGTGTTCAAGC    1787
NIID54          1688    AACATTAAATGATTTGGGCTATAAGACTGATGTGCGTGAGCTAAATTCAGGTGTTCAAGC    1747
NIID103         1734    AACATTAAATGATTTGGGCTATAAGACTGATGTGCGTGAGCTAAATTCAGGTGTTCAAGC    1793
NIID106         1682    AACATTAAATGATTTGGGCTATAAGACTGATGTGCGTGAGCTAAATTCAGGTGTTCAAGC    1741
                        * ***************************** ****************************

H44/76          1739    CATTATTATCGAGCCAAGCAGGCTGGTTGGTGGTGCCGATCCACGCCGAGAAGGTCGAGT    1798
H114/90         1739    CATTATTATCGAGCCAAGCAGGCTGGTTGGTGGTGCCGATCCACGCCGAGAAGGTCGAGT    1798
ATCC49226       1788    CATTATTATCGAGCCAAGCAGGCTGGTTGGTGGTGTCGATCCACGCCGAGAAGGTCGAGT    1847
NIID54          1748    CATTATTATCGAGCCAAGCAGGCTGGTTGGTGGTGTCGATCCACGCCGAGAAGGTCGAGT    1807
NIID103         1794    CATTATTATCGAGCCAAGCAGGCTGGTTGGTGGTGTCGATCCACGCCGAGAAGGTCGAGT    1853
NIID106         1742    CATTATTATCGAGCCAAGCAGGCTGGTTGGTGGTGTCGATCCACGCCGAGAAGGTCGAGT    1801
                        *********************************** ************************

H44/76          1799    GATGGGTGATTAG   1811
H114/90         1799    GATGGGTGATTAG   1811
ATCC49226       1848    GACGGGTGATTAG   1860
NIID54          1808    GACGGGTGATTAG   1820
NIID103         1854    GACGGGTGATTAG   1866
NIID106         1802    GACGGGTGATTAG   1814
                        ** **********
```
